# Association between dietary intake of fatty acids and colorectal cancer, a case-control study

**DOI:** 10.3389/fnut.2022.856408

**Published:** 2022-10-03

**Authors:** Soheila Shekari, Soroor Fathi, Zahra Roumi, Mohammad Esmail Akbari, Shirin Tajadod, Maryam Afsharfar, Naeemeh Hasanpour Ardekanizadeh, Fatemeh Bourbour, Seyed Ali Keshavarz, Mahtab Sotoudeh, Maryam Gholamalizadeh, Shiva Nemat Gorgani, Hanieh Shafaei Kachaei, Atiyeh Alizadeh, Saeid Doaei

**Affiliations:** ^1^Department of Nutrition, Science and Research Branch, Islamic Azad University, Tehran, Iran; ^2^Department of Community Nutrition, School of Nutrition and Food Science, Isfahan University of Medical Sciences, Isfahan, Iran; ^3^Department of Nutrition, Electronic Health and Statistics Surveillance Research Center, Science and Research Branch, Islamic Azad University, Tehran, Iran; ^4^Cancer Research Center, Shahid Beheshti University of Medical Sciences, Tehran, Iran; ^5^Department of Nutrition, School of Public Health, International Campus, Iran University of Medical Sciences, Tehran, Iran; ^6^Department of Nutrition, School of Medicine, Zahedan University of Medical Sciences, Zahedan, Iran; ^7^Department of Clinical Nutrition, School of Nutrition and Food Sciences, Shiraz University of Medical Sciences, Shiraz, Iran; ^8^Department of Clinical Nutrition and Dietetics, Faculty of Nutrition and Food Technology, National Nutrition and Food Technology Research Institute, Shahid Beheshti University of Medical Sciences, Tehran, Iran; ^9^Department of Clinical Nutrition, School of Nutritional Sciences and Dietetic, Tehran University of Medical Sciences, Tehran, Iran; ^10^Department of Clinical Nutrition and Dietetics, Research Institute, Shahid Beheshti University of Medical Science, Tehran, Iran; ^11^School of Nursing and Midwifery, Guilan University of Medical Sciences, Rasht, Iran; ^12^Department of Pharmacognosy, Faculty of Pharmacy, Tehran University of Medical Science, Tehran, Iran; ^13^Department of Community Nutrition, Faculty of Nutrition and Food Technology, National Nutrition and Food Technology Research Institute, Shahid Beheshti University of Medical Sciences, Tehran, Iran

**Keywords:** dietary intake, fatty acids, colorectal cancer risk, fat, cancer

## Abstract

**Background:**

The association of dietary fat and colorectal cancer (CRC) was frequently reported. However, few studies assessed the effects of different types of dietary fats on CRC. This study aimed to investigate the association between intakes of different types of dietary fatty acids with colorectal cancer risk.

**Methods:**

This case-control study was conducted on 480 participants including 160 CRC cases and 320 healthy controls in Firoozgar Hospital, Tehran, Iran. The intake of dietary fatty acids of the participants was assessed using a semi quantitative food frequency questionnaire (FFQ).

**Results:**

The mean intake of cholesterol (273.07 ± 53.63 vs. 254.17 ± 61.12, *P* = 0.001), polyunsaturated fatty acids (PUFA) (16.54 ± 4.20 vs. 15.41 ± 4.44, *P* = 0.012), and calorie (2,568.76 ± 404.48 vs. 2,493.38 ± 176.03, *P* = 0.006) was higher and the mean intake of oleic acid (5.59 ± 3.17 vs. 8.21 ± 5.46) and linoleic acid (6.03 ± 3.44 vs. 7.02 ± 4.08, *P* = 0.01) was lower in the case group compared to the control group. An inverse association was found between colorectal cancer (CRC) and dietary intake of oleic acid (OR: 0.85, CI 95% 0.80–0.90, *P* = 0.001), linoleic acid (OR: 0.85, CI 95% 0.78–0.93, *P* = 0.001), and α-linolenic acid (OR: 0.75, CI 95% 0.57–0.98, *P* = 0.04). The association remained significant after adjusting for age and sex, sleep, smoking, and alcohol consumption, and BMI.

**Conclusions:**

The results of this study support a protective effect of oleic acid, linoleic acid, and α-linolenic acid against CRC. Further longitudinal studies are warranted to confirm these results.

## Introduction

Colorectal cancer is the second leading cause of cancer death and the third most common cancer in the world. Keum and Giovannucci (1) Over the past 27 years, the incidence cases of CRC have doubled worldwide Keum and Giovannucci (1) and its incidence is steadily increasing in developing countries, with 2 million new cases in 2018 (1, 2). More than 700,000 people die annually due to CRC (3). The highest incidence of colorectal cancer was reported in North America, Australia, New Zealand, Western Europe, and Japan. Moreover, the prevalence of CRC has been increasing rapidly in Asian countries, including South Korea and Iran, in recent decades (3). Cancer is the third most common mortality factor in Iran after cardiovascular diseases and accidents. Also, findings show that colorectal cancer is the third most common cancer in Iranian men and fourth most common among Iranian women (4). CRC develops through the synergistic effects of several genetic and epigenetic factors that lead to transformation of the normal intestinal epithelium into invasive adenocarcinoma (5, 6). In order to reduce the mortality rate from colorectal cancer, prevention and early detection are key strategies (7). Reducing the incidence and mortality of CRC requires concerted efforts to reduce modifiable risk factors, and promote population-wide and targeted screening (1). Some prevalent risk factors for colorectal cancer include inflammatory bowel diseases such as Crohn's disease and ulcerative colitis, family history, genetics, and lifestyle factors such as lack of physical exercise, smoking, alcohol consumption, high fat and low fiber diets, high consumption of processed meats, and obesity (8–11). Among the factors associated with the development of CRC, modifiable risk factors such as unhealthy dietary intake, lack of physical exercise, alcohol consumption and smoking are of great importance 2018.

The effect of dietary intake on chronic diseases was frequently reported (12–15). Also, the association between colorectal cancer risk and diet has been studied in many studies (16–18). The findings show that the global spread of unhealthy, westernized diets high in red, processed meat and saturated fats (19). Epidemiological studies suggest that dietary fats may play important roles in colorectal cancer (20). Dietary fats can change the structure of the gut microbiota community and affect its function by modulating the production of metabolites (21). On the other hand, there is mounting evidence that consumption of excess dietary fats can enhance intestinal permeability differentially and induces IEC oxidative stress and apoptosis. In addition, a high-fat diet (HFD) enhances intestinal permeability directly by stimulating pro-inflammatory signaling cascades and indirectly *via* increasing TNFα, interleukin (IL) 1B, IL6, and interferon γ (IFNγ) and decreasing IL10, IL17, and IL22 (22). For example, omega-6 fatty acids are precursor to the production of inflammatory eicosanoids (23) and can be converted into various pro-inflammatory mediators such as prostaglandin E2 by the cyclooxygenase pathway (24). In addition, up-regulation of cyclooxygenase 2, which is involved in fatty acids metabolism, occurs in 50% of colon adenomas and 85% of colon cancers (25). Recent evidence indicates that PUFAs, such as docosahexaenoic acid (DHA), and eicosapentaenoic acid (EPA) can significantly affect the epigenome status of cells (26). It was also established that in CRC cells, PUFAs may affect the activity of certain microRNAs through alteration in promoter DNA methylation (27). An increased risk of colorectal cancer has been found to be associated with high dietary intake of n-6 Polyunsaturated fatty acids (PUFAs) such as arachidonic acid (28). On the other hand, Among specific types of PUFA, the preventive effects of total n-3 PUFA and marine-derived n-3 PUFA on CRC risk was reported (29). A prospective study reported that fish consumption protected women from colorectal cancer (30). A Case-control study on French Canadians identified an inverse association between dietary intake of n-3 PUFA and colorectal cancer (31). Furthermore, high saturated fatty acids intake may increase colorectal cancer risk (32). However, some studies reported contradictory results and found no associations of dietary fat or fatty acids with the risk of CRC (33–40). For example, a cohort study on dietary intake of fatty acids reported that there was no association between fatty acid intake and colorectal cancer risk (41). A systematic review of research papers published since the 1990s reported that there is no significant association between CRC risk with total fat intake, SFAs, MUFAs, and PUFAs (42). However, in Iran, so far, no study has been conducted on the relationship between dietary fats and the risk of colon cancer. So, the present case-control study aimed to investigate the association between intakes of different types of dietary fatty acids with colorectal cancer risk in Iranian adults.

## Methods and materials

### Study population

This case-control study was conducted from July 2020 to May 2021 on 480 randomly selected participants using the simple random sampling method including 160 patients with stage 3 and 4 colorectal cancer as the case group and 320 healthy people as the control group in Firoozgar Hospital in Tehran, Iran. The sample size was calculated using OpenEPI software (http://www.openepi.com/) and the odds ratios obtained in previous studies (43). The age-matched control group was selected from non-colorectal cancer patients referred to Firoozgar hospital for general check-ups. The inclusion criteria for the cases included histologically confirmed colorectal cancer by pathology, consent to participate in the study, no more than 3 months after diagnosis of cancer, age between 35 and 70 years, absence of other diseases affecting food intake, and not treated with drugs that affect food intake. Inclusion criteria for the control group included consent to participate in the study, age between 35 to 70 years, no history of cancer or other diseases that affect dietary intake, and no use of drugs that affect dietary intake.

### Data collection

Data of anthropometric measurements including weight, height, body mass index, and data on socio-demographic factors including age, sex, education, income level, marital status, health status, smoking status, and alcohol consumption were collected using a general questionnaire. All participants were assessed for dietary intake and physical activity through a face-to face interview with a trained researcher as follows.

### Dietary assessment

The dietary intake of the participants in the study was assessed using a validated semi-quantitative food frequency questionnaire (FFQ) (44). Data collected from FFQ were converted to grams using household measures and were analyzed using Nutritionist IV software (Sousa). The intake of total fat, saturated fatty acids, monounsaturated fatty acids, polyunsaturated fatty acids, linoleic acid, α-linolenic acid, eicosapentaenoic acid (EPA), and docosahexaenoic acid (DHA) during the year before diagnosis was assessed.

### Assessment of physical activity

Information on the level of physical activity was collected by the International Physical Activity Questionnaire (IPAQ), which has already been validated in Iran (45). Using this questionnaire, the amount of activity of people at home, at work, and during exercise was determined. Individuals were evaluated and compared in terms of activity using the metabolic equivalent of task (MET).

### Statistical analysis

Chi-square and independent *t*-test were used to compare qualitative and quantitative variables between the case and control groups, respectively. The logistic regression analysis method was used to investigate the relationship between CRC and dietary fatty acids. A stepwise (forward) selection procedure was used for modeling and variables were selected based on significance and background knowledge as crude regression model (Model 1), adjusted for age and sex (Model 2), further adjustment for physical activity, smoking, and alcohol consumption (Model 3), and additionally adjustment for BMI (Model 4). Statistical analyses were performed using SPSS software version 20 (SPSS Inc., Chicago, USA) and P-value < 0.05 was considered as significant in all analyses.

## Results

Distribution of the characteristics of the cases and controls is presented in Table 1. The cases had higher height (158.49 ± 7.59 vs. 156.11 ± 5.31 cm, *P* < 0.001), lower body mass index (BMI) (27.58 ± 3.25 vs. 28.76 ± 3.97 kg/m^2^, *P* < 0.001), and higher smoking (7.6 vs. 0.8%, *P* = 0.001). The amount of dietary fat intake of the participants is presented in Table 2. The mean intake of cholesterol (273.07 ± 53.63 vs. 254.17 ± 61.12 mg/d, *P* = 0.001), PUFA (16.54 ± 4.20 vs. 15.41 ± 4.44 g/d, *P* = 0.012), and calorie (2,568.76 ± 404.48 vs. 2,493.38 ± 176.03 kcal/d, *P* = 0.006) in the case group was significantly higher than the control group. The mean intake of oleic acid (5.59 ± 3.17 vs. 8.21 ± 5.46 g/d), linoleic acid (6.03 ± 3.44 vs. 7.02 ± 4.08 g/d, *P* = 0.01), and α-linolenic acid (0.83 ± 0.80 vs. 0.99 ± 0.73 g/d, *P* = 0.04) in the case group was significantly lower than the control group.

**Table 1:** Characteristics of the participants.

***Variable***	***Cases (n = 160)***	***Controls (n = 320)***	***P***
Age (yr)	52.36 ± 17.06	51.8 ± 10.82	0.05
Males (*n*, %)	81 (54/7%)	163 (51.1%)	0.11
Weight (kg)	69.39 ± 8.64	70.12 ± 10.59	0.44
Height (Cm)	158.49 ± 7.59	156.11 ± 5.31	<0.001
BMI (kg/m^2^)	27.58 ± 3.25	28.76 ± 3.97	<0.001
Physical activity (hr/wk)	7.51 ± 1.95	7.31 ± 1.58	0.28
Smoking (*n*, %)	2 (7.6%)	1 (0.8%)	0.003
Alcohol use (*n*, %)	0 (0.0%)	3 (1.2%)	0.73

**Table 2:** Dietary fat intake among the colorectal cancer patients and the control groups.

**Variable**	**Cases (*n* = 160)**	**Controls (*n* = 320)**	**P**
Calorie (kcal)	2,568.76 (± 404.48)	2,493.38 (±176.03)	0.006
Protein (g/d)	85.77 ± 9.16	85.39 ± 19.85	0.78
Fat (g/d)	88.90 ± 10.62	90.75 ± 20.15	0.20
Cholesterol (g/d)	273.07 ± 53.63	254.17 ± 61.12	0.001
SFA (g/d)	31.68 ± 10.04	31.19 ± 6.05	0.52
MUFA (g/d)	28.17 ± 4.83	28.79 ± 6.38	0.26
PUFA (g/d)	16.54 ± 4.20	15.41 ± 4.44	0.012
Oleic acid (mg/d)	5.59 ± 3.17	8.21 ± 5.46	0.001
Linoleic acid (mg/d)	6.03 ± 3.44	7.02 ± 4.08	0.01
α-linolenic acid (mg/d)	0.83 ± 0.80	0.99 ± 0.73	0.04
EPA (mg/d)	0.02 ± 0.02	0.02 ± 0.02	0.39
DHA (mg/d)	0.068 ± 0.05	0.063 ± 0.05	0.42

SFA, Saturated fatty acids; MUFA, Monounsaturated fatty acids; PUFA, Polyunsaturated fatty acids; EPA, Eicosapentaenoic acid; DHA, Docosahexaenoic acid.

The association between dietary intake of fatty acid and the risk of colorectal cancer is presented in Table 3. A negative association was found between CRC and dietary intake of linoleic acid (OR: 0.85, CI 95% 0.78–0.93, *P* = 0.001), oleic acid (OR: 0.85, CI 95% 0.80–0.90, *P* = 0.001), and α-linolenic acid (OR: 0.75, CI 95% 0.57–0.98, *P* = 0.04). The association remained significant after adjusting for age and sex (Model 2) and further adjustments for physical activity, smoking, and alcohol consumption (Model3). The association between CRC and oleic acid was disappeared after additional adjustment for BMI (Model 4).

**Table 3:** Logistic regression of the association between colorectal cancer and fat intake.

	Model 1*		**Model 2**		**Model 3**		**Model 4**	
	**OR (CI95%)**	***P***	**OR (CI95%)**	***P***	**OR (CI95%)**	***P***	**OR (CI95%)**	***P***
Cholesterol (g/d)	0.99 (0.99–0.99)	0.5	0.99 (0.99–1.0)	0.5	0.99 (0.98–0.99)	0.9	0.99 (0.98–0.99)	0.2
PUFA (g/d)	1.07 (1.01–1.13)	0.11	0.087 (0.05–0.14)	0.1	0.001 (0.001–0.001)	0.99	0.001 (0.001–0.001)	0.99
Oleic (mg/d)	0.85 (0.80–0.90)	0.001	0.53 (0.41–0.70)	0.001	0.51 (0.37–0.71)	0.001	0.001 (0.001–133,091.1)	0.43
Linoleic (mg/d)	0.85 (0.78–0.93)	0.001	0.89 (0.81–0.97)	0.014	0.89 (0.81–0.98)	0.02	0.85 (0.74–0.99)	0.04
α-linolenic acid (mg/d)	0.753 (0.57–0.98)	0.04	0.76 (0.57–0.99)	0.04	0.02 (0.01–0.07)	0.01	0.01 (0.01–0.07)	0.01

PUFA, Polyunsaturated fatty acids. Model 1: Crude, Model 2: Adjusted for sex and age. Model 3: Additionally adjusted for physical activity, tobacco, and alcohol level. Model 4: additionally adjusted for BMI.

## Discussion

The results of the present study indicated that there was an inverse association between colorectal cancer and dietary intake of oleic acid, linoleic acid and, α-linolenic acid. Epidemiological and experimental studies reported contradictory results on the association of dietary fatty acids and colorectal cancer. Butler et al. performed a nested case-control study of 350 colorectal cancer cases and an equal number of individually matched control subjects within the Singapore Chinese Health cohort Study to evaluate the associations between plasma fatty acid composition and colorectal cancer risk and reported a statistically significant inverse associations with colon cancer risk for higher levels of the essential PUFAs (i.e., α-linolenic and linoleic acids), oleic acid, and also found that the oleic: stearic acid ratio was positively associated with reduced frequency of colon cancer. The protective effects of alpha-linolenic acid may be due to its role as a precursor to the biosynthesis of EPA and DHA and their effects on increasing apoptosis and reducing the proliferation of cancer cells (46, 47).

Regarding the association of CRC with oleic acid, the consumption of an olive oil rich diet has been linked to a reduction in the incidence of cardiovascular disease and cancer (38), which was in line with the present study. Carrillo et al. reported that an oleic acid-enriched diet improved animal survival with lung tumor and confirm the anti-cancer properties of oleic acid (49). On the other hand, Bull et al. found that a high olive oil diet stimulated cervical cancer carcinogenesis, and oleic acid was considered to be associated with migration and invasion of cancer cells (58). Some experimental findings suggest that oleic acid consumption may induce colorectal cancer (CRC) whereas extra virgin olive oil minor compounds modulate mitogenic action of oleic acid on colon cancer and in this way, it has protective effects against this cancer (48). The exact mechanism of the association between cancer and oleic acid is not yet clear. Some studies reported an inhibition in cell proliferation induced by oleic acid in different tumor cell lines (49, 50). Oleic acid may suppress the over-expression of HER2 (erbB-2), an oncogene which plays a key role in the invasive progression and metastasis in several human cancers. In addition, oleic acid may induce apoptosis in carcinoma cells through increase in intracellular ROS production or caspase 3 activity (49). Oleic acid may induce NADPH oxidase 4 (NOX4) expression, resulting in an elevation of reactive oxygen species levels that can lead to promoted CRC metastasis (51). While, Carolina E. Storniolo et al., reported that the consumption of seed oils, high oleic acid seed oils, or olive oil will probably have different effects on CRC.

The results of the present study indicated an inverse association between CRC and omega-6 fatty acids. A cohort study in the United States observed no association between intake of omega-6 and omega-3 fatty acids and colorectal cancer (52). Another study in Canada found no association between omega-6 PUFA intake and colorectal cancer risk. The reason for these different results may be the different biological functions of different types of omega-6 fatty acids (53). For example, linoleic acid and arachidonic acid are both omega-6 fatty acids that may have different effects on carcinogenesis (54–56). One study reported a positive association with colon cancer for the desaturase indices reflecting increased synthesis of arachidonic acid (57). Eicosanoids derived from omega-6 may enhance the risk of CRC through production of pro inflammatory factors (58). For example, arachidonic acid is converted to proinflammatory factors such as prostaglandin E2 through the cyclooxygenase pathway and higher serum levels of prostaglandin E2 may promote carcinogenesis (24). However, some studies have indicated that some derivatives of linoleic acid may have anticancer effects. 15-LOX-1 is the principle enzyme for converting colonic linoleic acid into 13-S-hydroxyoctadecadienoic acid (13-S-HODE), which induces apoptosis (59). Additionally, peroxisome proliferator activated receptors (PPARs) are nuclear receptors for linoleic acid and arachidonic acid metabolites which increase colonic tumorigenesis. In a recent study 13-S-HODE was reported to bind to PPAR, inhibit its activation and decrease the expression of PPAR in CRC cells (58). These derivatives regulate a number of critical cancer-related signaling pathways through their effects on the activity of transcription factors, estrogen metabolism, lipid peroxidation, gene expression, insulin sensitivity, membrane fluid, cell proliferation, apoptosis, angiogenesis, and metastasis (58). Therefore, examining the association of fatty acids with colorectal cancer in terms of omega classification may be misleading. Moreover, several genetic, demographic, and environmental factors may influence the association between fatty acids and colorectal cancer (53). Cheng et al. reported that PUFA omega-6 was associated with increased risk of CRC in women and decreased CRC risk in men (60).

We found an inverse association between CRC and the intake of omega-3 fatty acids. Omega-3 may be a protective factor against CRC by reducing the level of pro-inflammatory cytokines produced by the cyclooxygenase pathway. The inefficient metabolic conversion of ALA to very long-chain omega-3 fatty acids including EPA and DHA (61) can be linked to adverse health outcomes (62). Several studies indicated that omega-3 PUFAs are crucial for inhibiting various types of tumors (33, 63). Angiogenesis mediators such as vascular endothelial growth factor (VEGF), platelet-derived growth factor (PDGF), and prostaglandin E2 (PGE2) may be inhibited by omega-3 PUFAs (46).

However, some other studies reported contradictory results. One study on Chinese women found no association between omega-3 intake and colorectal cancer (28). In addition, Reddy et al. found that intake of ALA omega-3 fatty acids was associated with an increased risk of CRC in women (64). Future studies on the effects of omega-3 fatty acids on CRC are required.

The strengths of this study were the adequate sample size, focus on diet prior to diagnosis, and highlighting the differences between different types of dietary fatty acids. However, this study had some limitations. One limitation of assessing dietary intake in case-control studies is the using of self-report FFQ which has potentially a recall bias. In addition, there are several other factors that may affect the results. However, the effects of some confounders such as age, sex, smoking, tobacco, and BMI were adjusted, but the effects of other factors, such as genetics and stress were not assessed.

## Conclusion

In the present study, the patients with CRC had higher intake of cholesterol and lower intake of oleic acid, linoleic acid, and α-linolenic acid than the control group. Higher intake of oleic acid, linoleic acid, and α-linolenic acid was associated with a reduced frequency of CRC. Dietary modification in terms of receiving rich sources of unsaturated fatty acids may be effective in reducing the risk of colorectal cancer. Further longitudinal studies are warranted to examine the effects of different types of fatty acids on colorectal cancer and to discover the underlying mechanisms.

## Data availability statement

The original contributions presented in the study are included in the article/supplementary material, further inquiries can be directed to the corresponding author.

## Ethics statement

The studies involving human participants were reviewed and approved by (Code: IR.SBMU.CRC.REC.1398.028). The patients/participants provided their written informed consent to participate in this study.

## Author contributions

SS, SF, ST, NH, ZR, and MG designed the study and carried out the data collection. MA, MEA, FB, SK, HS, MS, SN, AA, and SD were involved in the design of the study, analysis of the data, and critically reviewed the manuscript. All authors contributed to the article and approved the submitted version.

## Funding

Funding for this study was provided by Cancer Research Center (CRC), Shahid Beheshti University of Medical Sciences, Tehran, Iran (Code:15784).

## Conflict of interest

The authors declare that the research was conducted in the absence of any commercial or financial relationships that could be construed as a potential conflict of interest.

## Publisher's note

All claims expressed in this article are solely those of the authors and do not necessarily represent those of their affiliated organizations, or those of the publisher, the editors and the reviewers. Any product that may be evaluated in this article, or claim that may be made by its manufacturer, is not guaranteed or endorsed by the publisher.
